# Fertility preservation and the terminally ill patient: A case report with considerations for practice

**DOI:** 10.1017/S1478951526103150

**Published:** 2026-07-15

**Authors:** Tracey Norling, Maureen Tapfield, Kristen Ranse

**Affiliations:** 1 Specialist Palliative Care, Gold Coast Hospital and Health Service, Gold Coast, Australia; 2 Cancer and Palliative Care Outcomes Centre, Queensland University of Technology Faculty of Health, Kelvin Grove, Australia

**Keywords:** Fertility preservation, palliative care, communication, terminal care, family planning

## Abstract

**Objective:**

To raise awareness of fertility preservation and counseling in palliative care and explore considerations for practice.

**Methods:**

This case report describes the rapid decline and death of a 36-year-old man with astrocytoma. Despite early fertility discussions with the palliative care team, sudden clinical deterioration prevented semen collection prior to death. Following death, the spouse requested post-death sperm retrieval.

**Results:**

Post-death sperm retrieval and cryopreservation were successfully completed within the viability window. To achieve this, urgent interdisciplinary coordination across palliative care, emergency medicine, reproductive specialists, and hospital legal and executive teams was required.

**Significance of results:**

The case highlights the importance of early and ongoing fertility counseling for patients of reproductive age in palliative care. It demonstrates that clear pathways and coordinated systems can enable post-death sperm retrieval when aligned with patient and partner wishes. The development of evidence-based policies, training, and patient resources may reduce barriers and support clinicians to conduct sensitive, informed fertility discussions.

## Introduction

Palliative care seeks to support quality of life and a dignified death through holistic, person-centered care. For younger adults facing terminal illness, reproductive planning may be deeply tied to identity, legacy, and family well-being (Letourneau et al. 2012; Deshpande et al. 2015; Sharpless et al. 2021). While fertility counseling is recommended at cancer diagnosis, it is not consistently revisited as illness progresses, and patients frequently report unmet information needs (Letourneau et al. 2012; Deshpande et al. 2015; Sharpless et al. 2021; Jones et al. 2023). Barriers include perceived inappropriateness in the context of poor prognosis, uncertainty about responsibility, limited clinician knowledge, cost concerns, and personal discomfort discussing fertility (Letourneau et al. 2012; Deshpande et al. 2015; Winterling et al. 2020; Kieu et al. 2022). Addressing these barriers can support coping, reduce decisional regret, and align care with patient values (Letourneau et al. 2012; Deshpande et al. 2015). This case demonstrates how sustained, sensitive conversations and coordinated systems can enable reproductive options – including post-death sperm retrieval – when living donation is no longer feasible.

This case report is written in accordance with the CARE reporting guidelines (Riley et al. 2017) and was submitted to the Hospital Human Research Ethics Committee and deemed exempt as research (EX/2025/QGC/116683). The family consented to the publication of de-identified information.

## Case description

**Figure 1.**
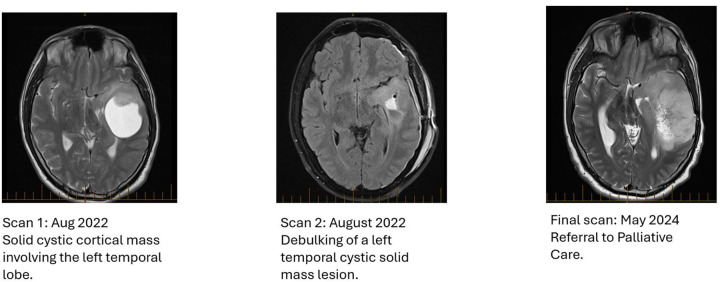
Neuroimaging scans.

**Figure 2.**
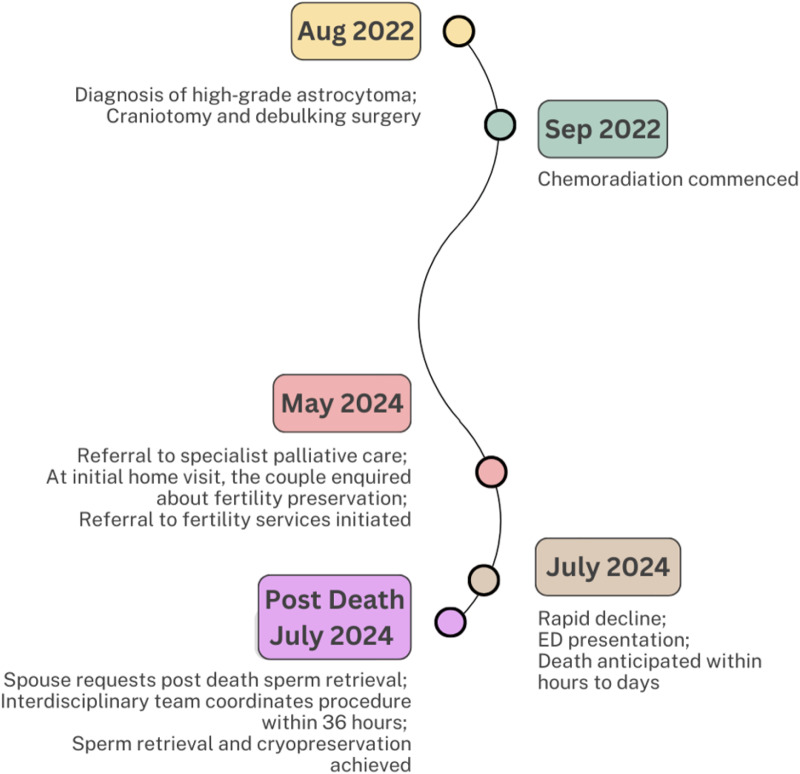
Timeline.

A 36-year-old man, partner and father of one child, presented to his General Practitioner in August 2022 with headaches. Initial imaging revealed a solid–cystic cortical mass in the left temporal lobe, and histopathology confirmed an Isocitrate Dehydrogenase (IDH)-mutant astrocytoma (WHO Grade 4) with O⁶-Methylguanine-DNA Methyltransferase (MGMT) unmethylated status. Diagnosis and monitoring relied on neuroimaging (see Figure 1) and clinical assessment. Initial management included surgical debulking and chemoradiation treatments with temporary disease control. However, disease progression prompted referral to specialist palliative care in May 2024 (see Figure 2). At the first consultation, the patient and partner expressed a desire to expand their family and requested fertility preservation information. With no history of infertility, fertility goals became central to care planning, and referral for semen collection and storage was initiated.

In July 2024, the patient experienced a rapid functional decline with a reduced Glasgow Coma Scale score that prompted presentation to the Emergency Department of an acute care hospital. Clinical review of the patient identified that he was in the last hours or days of life. His decline prevented semen collection. Following death, his spouse requested post-death sperm retrieval.

Challenges pertinent to this case were logistical, ethical, and legal, rather than laboratory: ensuring the appropriate conversations, authorizations, and processes for time-sensitive sperm retrieval after death. An interdisciplinary response of palliative care clinicians, emergency staff, reproductive specialists, social work, hospital legal counsel, and executive leadership enabled retrieval within the viability window commonly cited as ≤24–36 hours (Queensland Health 2024), with subsequent cryostorage according to reproductive medicine protocols. The immediate outcome was successful retrieval and cryopreservation. Longer-term reproductive outcomes (e.g., fertilization, embryo transfer, and pregnancy) were not available at the time of writing.

The partner stated: “We had always planned to grow our family. Knowing there is a possibility for future family planning brings comfort.” The partner’s feedback on the experience of the post-death sperm retrieval process emphasized the need for discretion (e.g., thoughtful bedside handover when multiple relatives are present) and clear written materials to support shared decision-making.

## Discussion

This case demonstrated that post-death sperm retrieval was feasible in one acute care hospital when underpinned by clear authorization pathways, rapid logistics, and psychosocial support. It highlighted key considerations, including legal frameworks, ethical issues, fertility counseling, interdisciplinary collaboration, and the need for training and support for clinicians.

The legality of posthumous reproduction, including post-death sperm retrieval, varies internationally and within Australia, where regulation is complex and state-specific (Ovics et al. 2022; Queensland Health 2024; Donigan et al. 2026). In Queensland, Australia, the Assisted Reproductive Technology Act 2024 (ART Act) governs the retrieval of gametes from a deceased or unresponsive person, while the Assisted Reproductive Technology Regulation 2026 details processes to authorize subsequent use of the retrieved gametes (Queensland Health 2024, 2026). Requests to retrieve gametes typically involve the spouse and consideration of known patient preferences, that is, the deceased or unresponsive patient previously consented or did not overtly object and would likely agree to retrieval. While not a legal analysis, the case underscores the importance of having local guidelines and procedures in place that comply with legislation (Ovics et al. 2022) and support patients and clinicians. These guidelines should include early liaison with hospital legal teams and reproductive specialists; the importance of documenting patient wishes and rapid escalation pathways; as well as procedures that meet viability constraints regarding post-death sperm retrieval (Donigan et al. 2026).

Post-death sperm retrieval raises ethical considerations for clinicians, with the principle of autonomy being a key consideration (Lawson et al. 2016; Donigan et al. 2026). In this case, it was evident that the patient’s partner had a desire for autonomy in her decision-making. Clinicians should ensure fertility counseling remains neutral to support autonomous decision-making without external pressure and without obligation to the deceased or other surviving extended family members (Sharpless et al. 2021; Jones et al. 2023). The request for retrieval of sperm and the subsequent use of the sperm are 2 discrete processes, and the patient and their partner should be supported to make informed decisions autonomously (Jones et al. 2023).

Ethical concerns related to the principles of beneficence and justice may also contribute to hesitancy amongst palliative care clinicians in relation to fertility preservation (Lawson et al. 2016). Concerns may include the patient’s poor prognosis, costs associated with fertility preservation, and worries about appropriateness (Letourneau et al. 2012; Winterling et al. 2020). In this case, the implication for practice is that clinicians should ensure both the patient and their partner have access to psychosocial support, acknowledging the moral complexity and associated grief. They should also be offered fertility preservation counseling where appropriate and desired.

Clinicians can use values-oriented prompts that explore fertility goals, such as “Have you completed your family?” or “Do you have questions about fertility preservation?” These questions can be integrated into routine assessments as prompts to normalize discussion, enable referral to fertility specialists as required, and support informed decision-making.

Fertility counseling should extend beyond diagnosis and be incorporated into standard palliative care for patients of reproductive age (Sharpless et al. 2021). Fertility counseling is associated with lower regret and improved coping for patients and their partners (Sharpless et al. 2021). In specialist palliative care, proactive and ongoing fertility preservation conversations can ensure that care is aligned with patient values and legacy goals (Letourneau et al. 2012; Deshpande et al. 2015). Ongoing fertility counseling can facilitate timely referral to reproductive specialists, increasing the likelihood of living donation, while allowing for contingency planning in the context of rapid clinical decline (Letourneau et al. 2012; Deshpande et al. 2015). These conversations can also prevent crisis-driven decision-making and the need for hastily navigated authorizations (Letourneau et al. 2012; Deshpande et al. 2015). Importantly, clear documentation of patient preferences, including consent or objection regarding post-death sperm retrieval, supports future decision-making and reduces ambiguity for families and clinicians during emotionally challenging circumstances.

Coordinated interdisciplinary care is critical. In this case, collaboration across clinical, legal, and administrative teams enabled timely post-death sperm retrieval and protected family privacy. Development of institutional policies and Standard Operating Procedures, including escalation pathways, contact lists, and documentation requirements, can improve coordination of practice, consistency, and efficiency (Donigan et al. 2026).

Clinician knowledge gaps remain a barrier (Sharpless et al. 2021; Kieu et al. 2022). Education that includes both fertility preservation and communication strategies can help improve clinician confidence. A brief educational intervention for cancer care nurses has been shown to increase awareness and confidence in initiating conversations about sex and fertility (Winterling et al. 2020), suggesting that similar training programs may also benefit palliative care nurses. For patients and their partners, written information sheets and educational resources (e.g., videos) that incorporate current fertility preservation guidelines, options for fertility preservation, associated costs, and local standard operating procedures may improve health literacy and support informed decision-making (Kieu et al. 2022).

## Strengths and limitations

This case offers a clear timeline, demonstrates coordinated interdisciplinary care, and highlights the role of fertility counseling in palliative care.

This case report is limited to a single case study from one health service in one jurisdiction in Australia; as such, legal frameworks and clinical policies will vary in other locations. Future work could evaluate protocolized retrieval pathways and patient-reported outcomes related to fertility counseling and decision-making in palliative care contexts.

## Conclusion

Fertility counseling is a valid component of specialist palliative care for patients of reproductive age. Early, values-based discussions and coordinated care can support quality of life, reduce decisional regret, and, when necessary, enable ethically conducted post-death sperm retrieval that is aligned with patient and family wishes.
